# Supplementary material for: Effects of blood urea nitrogen independent of the estimated glomerular filtration rate on the development of anemia in non-dialysis chronic kidney disease: The results of the KNOW-CKD study
Source: PLoS One. 2021 Sep 10;16(9):e0257305. doi: 10.1371/journal.pone.0257305 (PMC8432877; doi:10.1371/journal.pone.0257305)
Supplement: S1 Table — (DOCX) [file pone.0257305.s001.docx]

**S1 Table. Types and dose of diuretics according to BUN levels**

| Diuretics | | | | Total (N = 2,196) | Blood urea nitrogen | | | | *P*-value |
| --- | --- | --- | --- | --- | --- | --- | --- | --- | --- |
|  |  |  |  |  | 1st quartile | 2nd quartile | 3rd quartile | 4th quartile |  |
|  |  |  |  |  | (5.0-17.0 mg/dL) | (17.1-23.9 mg/dL) | (24.0-35.0 mg/dL) | (35.1-112.0 mg/dL) |  |
|  |  |  |  |  | (n = 572) | (n = 520) | (n = 557) | (n = 547) |  |
| ^†^Diuretics, n (%) | |  |  | 651 (30.3) | 70 (12.6) | 108 (21.4) | 198 (36.0) | 275 (50.9) | < 0.001 |
| Loop diuretics, n (%) | |  |  | 386 (17.6) | 17 (3.0) | 37 (7.1) | 126 (22.6) | 206 (37.7) | < 0.001 |
|  | Furosemide | n (%) |  | 340 (15.5) | 14 (2.4) | 29 (5.6) | 108 (19.4) | 189 (34.6) | < 0.001 |
|  |  | Categorical |  |  |  |  |  |  | <0.001 |
|  |  |  | 20 mg | 136 (6.2) | 6 (1.0) | 14 (2.7) | 49 (8.8) | 67 (12.2) |  |
|  |  |  | 40mg | 139 (6.3) | 6 (1.0) | 11 (2.1) | 41 (7.4) | 81 (14.8) |  |
|  |  |  | 60 or 80mg | 60 (2.7) | 2 (0.3) | 4 (0.8) | 17 (3.1) | 37 (6.8) |  |
|  |  |  | > 120mg | 5 (0.2) | 0 (0.0) | 0 (0.0) | 1 (0.2) | 4 (0.7) |  |
|  | Torasemide | n (%) |  | 46 (2.1) | 3 (0.5) | 8 (1.5) | 18 (3.2) | 17 (3.1) | 0.003 |
|  |  | Categorical |  |  |  |  |  |  | 0.011 |
|  |  |  | < 2.5 mg | 11 (0.5) | 0 (0.0) | 3 (0.3) | 4 (0.7) | 4 (0.7) |  |
|  |  |  | 5 mg | 28 (1.3) | 3 (0.5) | 4 (0.8) | 13 (2.3) | 8 (1.5) |  |
|  |  |  | 10 mg | 7 (0.7) | 0 (0.0) | 1 (0.2) | 1 (0.2) | 5 (0.9) |  |
| Distal tubular diuretics, n (%) | |  |  | 265 (12.1) | 53 (9.3) | 71 (13.7) | 72 (12.9) | 69 (12.6) | 0.111 |
|  | Hydrochlorothiazide | n (%) |  | 239 (10.9) | 49 (8.6) | 66 (12.7) | 63 (11.3) | 61 (11.2) | 0.167 |
|  |  | Categorical |  |  |  |  |  |  | 0.493 |
|  |  |  | 6.25 mg | 5 (0.2) | 2 (0.3) | 2 (0.4) | 1 (0.2) | 0 (0.0) |  |
|  |  |  | 12.5 mg | 201 (9.2) | 40 (7.0) | 56 (10.8) | 55 (9.9) | 50 (9.1) |  |
|  |  |  | 25 mg | 32 (1.5) | 7 (1.2) | 8 (1.5) | 7 (1.3) | 10 (1.8) |  |
|  |  |  | 50 mg | 1 (0.04) | 1 (0.04) | 0 (0.0) | 0 (0.0) | 0 (0.0) |  |
|  | Indapamide* | n (%) | 1.5mg | 18 (0.8) | 3 (0.5) | 4 (0.8) | 7 (1.3) | 4 (0.7) | 0.574 |
|  | Metolazone | n (%) |  | 8 (0.4) | 1 (0.2) | 1 (0.2) | 2 (0.4) | 4 (0.7) | 0.389 |
|  |  | Categorical |  |  |  |  |  |  | 0.670 |
|  |  |  | 2.5 mg | 3 (0.1) | 0 (0.0) | 1 (0.2) | 1 (0.2) | 1 (0.2) |  |
|  |  |  | 5 mg | 3 (0.1) | 1 (0.2) | 0 (0.0) | 0 (0.0) | 2 (0.4) |  |
|  |  |  | 10 mg | 2 (0.1) | 0 (0.0) | 0 (0.0) | 1 (0.2) | 1 (0.2) |  |

†Loop or distal tubule diuretics

*All patients had 1.5mg.
