# Supplementary material for: Effects of blood urea nitrogen independent of the estimated glomerular filtration rate on the development of anemia in non-dialysis chronic kidney disease: The results of the KNOW-CKD study
Source: PLoS One. 2021 Sep 10;16(9):e0257305. doi: 10.1371/journal.pone.0257305 (PMC8432877; doi:10.1371/journal.pone.0257305)
Supplement: S2 Table — (DOCX) [file pone.0257305.s002.docx]

**S2 Table. Multivariable linear regression analysis for related factors to BUN and BUN residual**

|  | **BUN** | | | **BUN residual** | | |
| --- | --- | --- | --- | --- | --- | --- |
|  | ***β*** | **95% CI** | ***P* value** | ***β*** | **95% CI** | ***P* value** |
| Age (year) | -0.01 | -0.07, 0.05 | 0.673 | 0.01 | -0.05, 0.07 | 0.758 |
| Sex (female) | -2.34 | -3.72, -0.96 | 0.001 | -1.92 | -3.24, -0.60 | 0.004 |
| DM (yes) | 0.11 | -1.34, 1.55 | 0.886 | 0.12 | -1.26, 1.50 | 0.719 |
| Smoking (Current or former) | 0.25 | -1.00, 1.50 | 0.697 | 0.22 | -0.98, 1.42 | 0.719 |
| Alcohol (yes) | 0.58 | -0.79, 1.95 | 0.406 | 0.59 | -0.72, 1.91 | 0.376 |
| Age adjusted CCI | -0.04 | -0.55, 0.48 | 0.886 | 0.07 | -0.42, 0.57 | 0.766 |
| eGFR (mL/min/1.73m^2^) | -0.23 | -0.30, -0.16 | <0.001 | 0.05 | -0.02, 0.12 | 0.132 |
| CKD stages Stage 1 | Reference |  |  | Reference |  |  |
| Stage 2 | -4.12 | -6.84, -1.40 | 0.003 | 1.14 | -1.46, 3.75 | 0.367 |
| Stage 3a | -5.16 | -9.14, -1.17 | 0.011 | 0.93 | -2.89, 4.75 | 0.633 |
| Stage 3b | -3.48 | -8.42, 1.46 | 0.167 | 0.28 | -4.45, 5.01 | 0.908 |
| Stage 4 | 4.16 | -1.70, 10.02 | 0.164 | 0.27 | -5.35, 5.88 | 0.926 |
| Stage 5 | 15.11 | 8.33, 21.89 | <0.001 | -3.23 | -9.73, 3.27 | 0.330 |
| Log (1000×DPI) | 23.54 | 18.99 28.10 | <0.001 | 24.47 | 20.11, .28.84 | <0.001 |
| Albumin (g/dL) | 0.11 | -1.25, 1.47 | 0.875 | 0.03 | -1.28, 1.33 | 0.969 |
| Phosphorus (mg/dL) | 4.36 | 3.52. 5.20 | <0.001 | 3.67 | 2.87, 4.48 | <0.001 |
| Uric acid (mg/dL) | 0.56 | 0.28, 0.85 | <0.001 | 0.64 | 0.37, 0.92 | <0.001 |
| Total CO_2_ (mmol/L) | -0.27 | -0.43, -0.11 | 0.001 | -0.17 | -0.32, -0.02 | 0.028 |
| Sodium (mmol/L) | -0.30 | -0.50, -0.10 | 0.004 | -0.32 | -0.51, -0.12 | 0.001 |
| Potassium (mmol/L) | 2.46 | 1.49, 3.44 | <0.001 | 2.29 | 1.36, 3.22 | <0.001 |
| Log UPCR | -0.27 | -1.20, 0.66 | 0.570 | -0.53 | -1.41, 0.36 | 0.245 |
| Log CRP | -0.25 | -1.01, 0.51 | 0.524 | -0.04 | -0.77, 0.69 | 0.921 |
| Log 24hr-urine volume | -7.39 | -10.77, -4.01 | <0.001 | -8.13 | -11.37, -4.88 | <0.001 |
| ACEi or ARB, n (%) | -0.77 | -2.18, 0.65 | 0.288 | -0.62 | -1.98, 0.73 | 0.367 |
| ^†^Diuretics, n (%) | 3.26 | 2.14, 4.38 | <0.001 | 2.90 | 1.83. 3.97 | <0.001 |
| Statin, n (%) | -1.61 | -2.59, -0.64 | 0.001 | -1.33 | -2.26, -0.39 | 0.005 |

BUN, blood urea nitrogen; DM, diabetes mellitus; CCI, Charlson comorbidity index; eGFR, estimated glomerular filtration rate as determined by the CKD-EPI creatinine equation; DPI, dietary protein intake; UPCR, urine protein creatinine ratio; CRP, C-reactive protein; ACEi, angiotensin converting enzyme inhibitor; ARB, angiotensin II receptor blocker

^†^Loop or distal tubule diuretics
