# Supplementary material for: Effects of blood urea nitrogen independent of the estimated glomerular filtration rate on the development of anemia in non-dialysis chronic kidney disease: The results of the KNOW-CKD study
Source: PLoS One. 2021 Sep 10;16(9):e0257305. doi: 10.1371/journal.pone.0257305 (PMC8432877; doi:10.1371/journal.pone.0257305)
Supplement: S3 Table — (DOCX) [file pone.0257305.s003.docx]

**S3 Table. BUN residual and DPI according to CKD stages**

| CKD stage | BUN residual | | DPI | |
| --- | --- | --- | --- | --- |
|  | Incident anemia (-) | Incident anemia (+) | Incident anemia (-) | Incident anemia (+) |
| Stage 1-2 (n = 609) | -0.23 ± 3.66 | -0.01 ± 4.17 | 1.05 (0.89, 1.25) | 1.05 (0.88, 1.23) |
| Stage 3 (n = 451) | -1.88 ± 4.85 | -0.90 ± 8.50 | 0.99 (0.85, 1.22) | 0.99 (0.85, 1.13) |
| Stage 4-5 (n = 109) | 0.16 ± 8.43 | -4.21 ± 8.95 | 0.98 (0.89, 1.03) | 0.96 (0.85, 1.12) |

BUN, blood urea nitrogen; DPI, dietary protein intake; CKD, chronic kidney disease
