# Supplementary material for: Effects of blood urea nitrogen independent of the estimated glomerular filtration rate on the development of anemia in non-dialysis chronic kidney disease: The results of the KNOW-CKD study
Source: PLoS One. 2021 Sep 10;16(9):e0257305. doi: 10.1371/journal.pone.0257305 (PMC8432877; doi:10.1371/journal.pone.0257305)
Supplement: S4 Table — (DOCX) [file pone.0257305.s004.docx]

**S4 Table. Types and dose of diuretics with respect to anemia development**

| Characteristics |  |  |  | Total (N = 1,169) | Groups | | *P*-value |
| --- | --- | --- | --- | --- | --- | --- | --- |
|  |  |  |  |  | Incident anemia (-) (n = 755) | Incident anemia (+) (n = 414) |  |
| ^†^Diuretics, n (%) | |  |  | 238 (20.4) | 132 (17.5) | 106 (25.6) | <0.001 |
| Loop diuretics, n (%) | |  |  | 101 (8.6) | 41 (5.4) | 60 (14.5) | <0.001 |
|  | Furosemide | n (%) |  | 81 (6.9) | 33 (4.4) | 48 (11.6) | <0.001 |
|  |  | Categorical |  |  |  |  | <0.001 |
|  |  |  | 20 mg | 40 (3.4) | 15 (2.0) | 25 (6.0) |  |
|  |  |  | 40mg | 32 (2.7) | 15 (2.0) | 17 (4.1) |  |
|  |  |  | 60 or 80mg | 8 (0.7) | 3 (0.4) | 5 (1.2) |  |
|  |  |  | > 120mg | 1 (0.1) | 0 (0.0) | 1 (0.2) |  |
|  | Torasemide | n (%) |  | 20 (1.7) | 8 (1.1) | 12 (2.9) | 0.020 |
|  |  | Categorical |  |  |  |  | 0.049 |
|  |  |  | < 2.5 mg | 6 (0.5) | 3 (0.4) | 3 (0.7) |  |
|  |  |  | 5 mg | 11 (0.9) | 5 (0.7) | 6 (1.4) |  |
|  |  |  | 10 mg | 3 (0.3) | 0 (0.0) | 3 (0.7) |  |
| Distal tubular diuretics, n (%) | |  |  | 137 (11.7) | 91 (12.1) | 46 (11.1) | 0.632 |
|  | Hydrochlorothiazide | n (%) |  | 130 (11.1) | 88 (11.7) | 42 (10.1) | 0.432 |
|  |  | Categorical |  |  |  |  | 0.786 |
|  |  |  | 6.25 mg | 3 (0.3) | 2 (0.3) | 1 (0.2) |  |
|  |  |  | 12.5 mg | 112 (9.6) | 77 (10.2) | 35 (8.5) |  |
|  |  |  | 25 mg | 15 (1.3) | 9 (1.2) | 6 (1.4) |  |
|  | Indapamide* | n (%) | 1.5mg | 4 (0.3) | 2 (0.3) | 2 (0.5) | 0.541 |
|  | Metolazone | n (%) |  | 3 (0.3) | 1 (0.1) | 2 (0.5) | 0.257 |
|  |  | Categorical |  |  |  |  | 0.366 |
|  |  |  | 2.5 mg | 2 (0.2) | 1 (0.1) | 1 (0.2) |  |
|  |  |  | 10 mg | 1 (0.1) | 0 (0.0) | 1 (0.2) |  |

†Loop or distal tubule diuretics

*All patients had 1.5mg.
