# Supplementary material for: Effects of blood urea nitrogen independent of the estimated glomerular filtration rate on the development of anemia in non-dialysis chronic kidney disease: The results of the KNOW-CKD study
Source: PLoS One. 2021 Sep 10;16(9):e0257305. doi: 10.1371/journal.pone.0257305 (PMC8432877; doi:10.1371/journal.pone.0257305)
Supplement: Attachment — Submitted filename: Reponse letter.docx [file pone.0257305.s005.docx]

**Point-by-Point Responses to the Reviewers**

**Additional Editor Comments**

***1.More information on BUN residual is needed in the introduction section.***

Response: Thank you for your comment.

We described in detail in the introduction section.

In line 113, “Introduction” paragraph page 6

Therefore, we hypothesized that BUN would have independent effects of eGFR on the development of anemia in NDCKD by representing the accumulation of uremic toxins which could inhibit effective erythropoiesis in NDCKD. ~~To minimize the confounding effects from the strong collinearities between BUN, eGFR, and anemia, BUN residuals obtained from the fitted curve between BUN and eGFR~~ ~~were used instead of BUN in all the models adjusted by eGFR [22.23].~~ Because both BUN and hemoglobin level in CKD are strongly influenced by renal function represented by eGFR, a parameter which excludes the effect of eGFR on BUN is needed to evaluate the effect of BUN independent of eGFR on anemia in CKD. Residual was proposed as one of the methods modeling the association of a specific constituent and outcome [22] similar to our study in which eGFR, volume status, protein intake are important constituents of BUN. BUN residual was actually used in a study to assess the independent association of eGFR between BUN and the development of diabetes mellitus [23]. Considering that the distribution of BUN is widely spread at the same eGFR [17], the authors calculate BUN residual to minimize the impact of kidney function on BUN levels. BUN residuals were obtained from the fitted curve between BUN and eGFR to minimize the confounding effects from the strong collinearities between BUN, eGFR, and anemia [22,23]. BUN residual may be an indicator that reflects factors affecting the BUN level except for the effects of eGFR, and in stable CKD patients, the count of protein intake will be the main factor in determining BUN residual.

***2.The criteria of ESA prescription should be mentioned in the method section.***

Response: Thank you for your comment.

ESA was considered for prescription under the discretion of physicians in participating hospitals in patients with hemoglobin < 10 g/dL in accordance with the KDIGO guidelines and started ESA in patients with eGFR < 30 mL/min/1.73m^2^ in accordance with the insurance coverage of Korea. During the use of ESA, physicians adjusted the dose of the ESA to maintain hemoglobin 10-11 g/dL (or hematocrit 30-33%) and stopped the ESA in hemoglobin > 11 g/dL (or hematocrit > 33%).

We added the criteria of ESA prescription in the method section

In line 181, “Methods: Clinical and laboratory measurements” paragraph page 9

Erythropoiesis-stimulating agent (ESA) was prescribed under the discretion of physicians in participating hospitals in patients with hemoglobin < 10 g/dL in accordance with the KDIGO guidelines [30], excluding other causes of anemia other than anemia of CKD. In addition, ESA was started in patients with eGFR < 30 mL/min/1.73m^2^ in accordance with the insurance coverage of Korea. During the use of ESA, physicians adjusted the dose of the ESA to maintain hemoglobin 10-11 g/dL (or hematocrit 30-33%) and stopped the ESA in hemoglobin > 11 g/dL (or hematocrit > 33%).

***3.The medications or conditions that potentially influence the BUN level should be considered in this study.***

Response: Thank you for your comment.

Our cohort excluded those with heart failure (NYHA class 3 or 4) or acute illness at the time of study enrollment. In addition, patients have mostly prescribed diuretics for blood pressure control rather than edema, and the actual dose of the diuretic was not so high. We revised and added supplementary S1 and S4 Table.

Because our study also excluded the patients with active glomerulonephritis, the use of corticosteroid which would increase BUN level was not sought and recorded.

In line 256, “Results: Baseline clinical characteristics of the study subjects” paragraph page 12

Subjects in the high BUN quartiles received more diuretics (*P* <0.001). Considering the types and dose of diuretics, the actual dose of the diuretics was not so high (S1 Table).

In line 350, “Results: Characteristics of patients who developed anemia” paragraph page 20

Subjects in the *de novo* anemia group received more diuretics (*P* <0.001). Considering the types and dose of diuretics, the actual dose of the diuretic was not so high (S4 Table).

**[Journal Requirements]**

***1). Please ensure that your manuscript meets PLOS ONE's style requirements, including those for file naming. The PLOS ONE style templates can be found at https://journals.plos.org/plosone/s/file?id=wjVg/PLOSOne_formatting_sample_main_body.pdf and***

***https://journals.plos.org/plosone/s/file?id=ba62/PLOSOne_formatting_sample_title_authors_affiliations.pdf***

Response: Thank you for your comment.

We rechecked PLOS ONE's style requirements and meet requirements.

***2). Please provide a sample size and power calculation in the Methods, or discuss the reasons for not performing one before study initiation.***

Response: Thank you for your valuable comment.

In the initial study design of this prospective cohort, we had aimed to enroll a total of 2,450 patients. This number was calculated to enroll about 600 subjects in each subgroup (glomerulonephritis, diabetic nephropathy, hypertensive nephropathy, and polycystic kidney disease) with 80% power achieved to detect hazard ratios of approximately 2.00 and 1.60 for exposure with the prevalence of 0.1 and 0.5 and a dropout rate of 10%. More details of the cohort design were previously published (Oh et al., BMC Nephrology 2014). As shown in Figure 1, a total of 2,238 subjects were recruited initially but the final analysis was conducted in 2,196 subjects, excluding patients who were not eligible for the analysis in this study (flow chart in Fig1).

We added in method section.

In line 142, “Methods: study design and population’ paragraph page 7

In the initial study design of this prospective cohort, the authors had aimed to enroll a total of 2,450 patients. This number was calculated to include approximately 600 subjects in each subgroup (diabetic nephropathy, hypertensive nephropathy, glomerulonephritis, and polycystic kidney disease) with 80% power achieved to detect hazard ratios of approximately 2.00 and 1.60 for exposure with prevalence of 0.1 and 0.5, respectively, and a dropout rate of 10%.

***3). Please note that PLOS does not permit references to “[data] not shown.” Authors should provide the relevant data within the manuscript, the Supporting Information files, or in a public repository. If the data are not a core part of the research study being presented, we ask that authors remove any references to these data.***

Response: Thank you for your comment.

We provided all the relevant data within the manuscript and the Supporting Information files.

***4). We noticed you have some minor occurrence of overlapping text with the following previous publication(s), which needs to be addressed:***

***- https://centerhealthyminds.org/assets/files-publications/goldberg-still-facial-photographs.pdf***

***- https://bmcnephrol.biomedcentral.com/articles/10.1186/s12882-018-1005-3***

***- https://www.nature.com/articles/s41598-019-52499-6***

***In your revision ensure you cite all your sources (including your own works), and quote or rephrase any duplicated text outside the methods section. Further consideration is dependent on these concerns being addressed.***

Response: Thank you for your comment.

-https://centerhealthyminds.org/assets/files-publications/goldberg-still-facial-photographs.pdf

: We did not refer to this paper and the content is completely different from our manuscript. Could you let us know the problem part?

- https://bmcnephrol.biomedcentral.com/articles/10.1186/s12882-018-1005-3

- https://www.nature.com/articles/s41598-019-52499-6

: The two papers were published using the KNOW-CKD cohort study data, in which the first author of this manuscript participated as co-author or first author. There are unique parts that cannot be changed in the method, such as hospital name or IRB number, so there may be some overlapping text. We modified the parts that can be changed as much as possible.

**Reviewer #1:**

***1. What is the clinical meaning of the BUN residual? The authors need to expand more details of BUN residual in the section of INTRODUCTION.***

Response: Thank you for your comment.

We described in detail in the introduction section.

In line 113, “Introduction” paragraph page 6

Therefore, we hypothesized that BUN would have independent effects of eGFR on the development of anemia in NDCKD by representing the accumulation of uremic toxins which could inhibit effective erythropoiesis in NDCKD. ~~To minimize the confounding effects from the strong collinearities between BUN, eGFR, and anemia, BUN residuals obtained from the fitted curve between BUN and eGFR~~ ~~were used instead of BUN in all the models adjusted by eGFR [22.23].~~ Because both BUN and hemoglobin level in CKD are strongly influenced by renal function represented by eGFR, a parameter which excludes the effect of eGFR on BUN is needed to evaluate the effect of BUN independent of eGFR on anemia in CKD. Residual was proposed as one of the methods modeling the association of a specific constituent and outcome [22] similar to our study in which eGFR, volume status, protein intake are important constituents of BUN. BUN residual was actually used in a study to assess the independent association of eGFR between BUN and the development of diabetes mellitus [23]. Considering that the distribution of BUN is widely spread at the same eGFR [17], the authors calculate BUN residual to minimize the impact of kidney function on BUN levels. BUN residuals were obtained from the fitted curve between BUN and eGFR to minimize the confounding effects from the strong collinearities between BUN, eGFR, and anemia [22,23]. BUN residual may be an indicator that reflects factors affecting the BUN level except for the effects of eGFR, and in stable CKD patients, the count of protein intake will be the main factor in determining BUN residual.

***2. The type and dose of diuretics are the major concern in this study. Higher diuretic dose would change the levels of BUN and BUN residual easily. How did the authors adjust this variable?***
Response: Thank you for your valuable comment.

We presented the type and dose of diuretics in S1 and S4 Table.

(In Initial Table 3, numbers of diuretics and statin were written opposite to each other, so we modified correctly.)

When the analysis was conducted in detail according to the types and dose of diuretics not just the use of diuretics, the results were similar in relation to BUN or BUN residual and hemoglobin or anemia development.

| **Multivariable linear regression analyses of the association between BUN, BUN residual, and hemoglobin level.** | | | | | | | | | |
| --- | --- | --- | --- | --- | --- | --- | --- | --- | --- |
|  | | ****β* (95% CI)** | ***P* value** | | | *****(β* (95% CI)** | ***P* value** | | |
| BUN | | -0.03 | <0.001 | | | -0.03 | <0.001 | | |
|  |  | (-0.04, -0.03) |  |  |  | (-0.04, -0.03) |  |  |  |
| BUN residual | | -0.03 | <0.001 | | | -0.03 | <0.001 | | |
|  |  | (-0.04, -0.02) |  |  |  | (-0.04, -0.02) |  |  |  |
| **Cox regression analysis of the relationship between BUN, BUN residual, and anemia development.** | | | | | | | | | |
|  | ***HR (95% CI)** | | | ***P* value** | ****HR (95% CI)** | | | ***P* value** |  |
| BUN | 1.02 (1.01, 1.04) | | | 0.002 | 1.02 (1.01, 1.04) | | | 0.002 |  |
| BUN residual | 1.02 (1.00, 1.04) | | | 0.031 | 1.02 (1.00, 1.04) | | | 0.036 |  |

*adjusted for age, sex, smoking, SBP, DM, HTN, CCI, eGFR, log UPCR, WBC, platelet, log CRP, albumin, total cholesterol, log ferritin, log hepcidin, iron, and, TSAT, iron replacement therapy, ESA, ACEi or ARB, statin, and **the presence of diuretics**

**adjusted for age, sex, smoking, SBP, DM, HTN, CCI, eGFR, log UPCR, WBC, platelet, log CRP, albumin, total cholesterol, log ferritin, log hepcidin, iron, and, TSAT, iron replacement therapy, ESA, ACEi or ARB, statin, and **diuretic groups according to types and dose**

**Diuretic groups were categorized as 0 to 4 using loop diuretics or distal tubule diuretics.

0: none

1: thiazide ≤ 12.5mg, indapamide ≤ 1.5mg, metolazone ≤ 2.5mg

2: thiazide > 12.5mg, indapamide > 1.5mg, metolazone > 2.5mg

3: furosemide ≤ 20mg, torasemide ≤ 10mg (The dosage of torasemide was up to 10 mg in our patients.)

4: furosemide > 20mg

Our cohort excluded those with heart failure (NYHA class 3 or 4) or acute illness at the time of registration. Patients used different diuretics according to the eGFR (distal tubule diuretics on higher eGFR patients and loop diuretics on lower eGFR patients) and have mostly prescribed diuretics for blood pressure control rather than edema, and the actual dose of the diuretic was not so high. Therefore, we thought that the types and dose of diuretics did not influence a significant change in BUN in our study.

In line 256, “Results: Baseline clinical characteristics of the study subjects” paragraph page 12

Subjects in the high BUN quartiles received more diuretics (*P* <0.001). Considering the types and dose of diuretics, the actual dose of the diuretics was not so high (S1 Table).

In line 350, “Results: Characteristics of patients who developed anemia” paragraph page 20

Subjects in the *de novo* anemia group received more diuretics (*P* <0.001). Considering the types and dose of diuretics, the actual dose of the diuretic was not so high (S4 Table).

In Supporting information

**S1 Table. Types and dose of diuretics according to BUN levels**

| Diuretics | | | | Total (N = 2,196) | Blood urea nitrogen | | | | *P*-value |
| --- | --- | --- | --- | --- | --- | --- | --- | --- | --- |
|  |  |  |  |  | 1st quartile | 2nd quartile | 3rd quartile | 4th quartile |  |
|  |  |  |  |  | (5.0-17.0 mg/dL) | (17.1-23.9 mg/dL) | (24.0-35.0 mg/dL) | (35.1-112.0 mg/dL) |  |
|  |  |  |  |  | (n = 572) | (n = 520) | (n = 557) | (n = 547) |  |
| ^†^Diuretics, n (%) |  |  |  | 651 (30.3) | 70 (12.6) | 108 (21.4) | 198 (36.0) | 275 (50.9) | < 0.001 |
| Loop diuretics |  |  |  | 386 (17.6) | 17 (3.0) | 37 (7.1) | 126 (22.6) | 206 (37.7) | < 0.001 |
|  | Furosemide | n (%) |  | 340 (15.5) | 14 (2.4) | 29 (5.6) | 108 (19.4) | 189 (34.6) | < 0.001 |
|  |  | Categorical |  |  |  |  |  |  | <0.001 |
|  |  |  | 20 mg | 136 (6.2) | 6 (1.0) | 14 (2.7) | 49 (8.8) | 67 (12.2) |  |
|  |  |  | 40mg | 139 (6.3) | 6 (1.0) | 11 (2.1) | 41 (7.4) | 81 (14.8) |  |
|  |  |  | 60 or 80mg | 60 (2.7) | 2 (0.3) | 4 (0.8) | 17 (3.1) | 37 (6.8) |  |
|  |  |  | > 120mg | 5 (0.2) | 0 (0.0) | 0 (0.0) | 1 (0.2) | 4 (0.7) |  |
|  | Torasemide | n (%) |  | 46 (2.1) | 3 (0.5) | 8 (1.5) | 18 (3.2) | 17 (3.1) | 0.003 |
|  |  | Categorical |  |  |  |  |  |  | 0.011 |
|  |  |  | < 2.5 mg | 11 (0.5) | 0 (0.0) | 3 (0.3) | 4 (0.7) | 4 (0.7) |  |
|  |  |  | 5 mg | 28 (1.3) | 3 (0.5) | 4 (0.8) | 13 (2.3) | 8 (1.5) |  |
|  |  |  | 10 mg | 7 (0.7) | 0 (0.0) | 1 (0.2) | 1 (0.2) | 5 (0.9) |  |
| Distal tubular diuretics |  |  |  | 265 (12.1) | 53 (9.3) | 71 (13.7) | 72 (12.9) | 69 (12.6) | 0.111 |
|  | Thiazide | n (%) |  | 239 (10.9) | 49 (8.6) | 66 (12.7) | 63 (11.3) | 61 (11.2) | 0.167 |
|  |  | Categorical |  |  |  |  |  |  | 0.493 |
|  |  |  | 6.25 mg | 5 (0.2) | 2 (0.3) | 2 (0.4) | 1 (0.2) | 0 (0.0) |  |
|  |  |  | 12.5 mg | 201 (9.2) | 40 (7.0) | 56 (10.8) | 55 (9.9) | 50 (9.1) |  |
|  |  |  | 25 mg | 32 (1.5) | 7 (1.2) | 8 (1.5) | 7 (1.3) | 10 (1.8) |  |
|  |  |  | 50 mg | 1 (0.04) | 1 (0.04) | 0 (0.0) | 0 (0.0) | 0 (0.0) |  |
|  | Indapamide* | n (%) | 1.5mg | 18 (0.8) | 3 (0.5) | 4 (0.8) | 7 (1.3) | 4 (0.7) | 0.574 |
|  | Metolazone | n (%) |  | 8 (0.4) | 1 (0.2) | 1 (0.2) | 2 (0.4) | 4 (0.7) | 0.389 |
|  |  | Categorical |  |  |  |  |  |  | 0.670 |
|  |  |  | 2.5 mg | 3 (0.1) | 0 (0.0) | 1 (0.2) | 1 (0.2) | 1 (0.2) |  |
|  |  |  | 5 mg | 3 (0.1) | 1 (0.2) | 0 (0.0) | 0 (0.0) | 2 (0.4) |  |
|  |  |  | 10 mg | 2 (0.1) | 0 (0.0) | 0 (0.0) | 1 (0.2) | 1 (0.2) |  |

†Loop or distal tubule diuretics

*All patients had 1.5mg.

**S4 Table. Types and dose of diuretics with respect to anemia development**

| Characteristics |  |  |  | Total (N = 1,169) | Groups | | *P*-value |
| --- | --- | --- | --- | --- | --- | --- | --- |
|  |  |  |  |  | Incident anemia (-) (n = 755) | Incident anemia (+) (n = 414) |  |
| ^†^Diuretics, n (%) |  |  |  | 238 (20.4) | 132 (17.5) | 106 (25.6) | <0.001 |
| Loop diuretics |  |  |  | 101 (8.6) | 41 (5.4) | 60 (14.5) | <0.001 |
|  | Furosemide | n (%) |  | 81 (6.9) | 33 (4.4) | 48 (11.6) | <0.001 |
|  |  | Categorical |  |  |  |  | <0.001 |
|  |  |  | 20 mg | 40 (3.4) | 15 (2.0) | 25 (6.0) |  |
|  |  |  | 40mg | 32 (2.7) | 15 (2.0) | 17 (4.1) |  |
|  |  |  | 60 or 80mg | 8 (0.7) | 3 (0.4) | 5 (1.2) |  |
|  |  |  | > 120mg | 1 (0.1) | 0 (0.0) | 1 (0.2) |  |
|  | Torasemide | n (%) |  | 20 (1.7) | 8 (1.1) | 12 (2.9) | 0.020 |
|  |  | Categorical |  |  |  |  | 0.049 |
|  |  |  | < 2.5 mg | 6 (0.5) | 3 (0.4) | 3 (0.7) |  |
|  |  |  | 5 mg | 11 (0.9) | 5 (0.7) | 6 (1.4) |  |
|  |  |  | 10 mg | 3 (0.3) | 0 (0.0) | 3 (0.7) |  |
| Distal tubular diuretics |  |  |  | 137 (11.7) | 91 (12.1) | 46 (11.1) | 0.632 |
|  | Thiazide | n (%) |  | 130 (11.1) | 88 (11.7) | 42 (10.1) | 0.432 |
|  |  | Categorical |  |  |  |  | 0.786 |
|  |  |  | 6.25 mg | 3 (0.3) | 2 (0.3) | 1 (0.2) |  |
|  |  |  | 12.5 mg | 112 (9.6) | 77 (10.2) | 35 (8.5) |  |
|  |  |  | 25 mg | 15 (1.3) | 9 (1.2) | 6 (1.4) |  |
|  | Indapamide* | n (%) | 1.5mg | 4 (0.3) | 2 (0.3) | 2 (0.5) | 0.541 |
|  | Metolazone | n (%) |  | 3 (0.3) | 1 (0.1) | 2 (0.5) | 0.257 |
|  |  | Categorical |  |  |  |  | 0.366 |
|  |  |  | 2.5 mg | 2 (0.2) | 1 (0.1) | 1 (0.2) |  |
|  |  |  | 10 mg | 1 (0.1) | 0 (0.0) | 1 (0.2) |  |

†Loop or distal tubule diuretics

*All patients had 1.5mg.

In line 318, “Result: Cross-sectional analysis of the association between BUN, BUN residual, and hemoglobin level” paragraph page 18

When adjustment was done for the types and dose of diuretics and not just the use of diuretics (group 1: none; group 2: thiazide ≤ 12.5 mg, indapamide ≤ 1.5 mg, metolazone ≤ 2.5 mg; group 3: thiazide > 12.5 mg, indapamide > 1.5 mg, metolazone > 2.5 mg; group 4: furosemide ≤ 20 mg, torasemide ≤ 10 mg; group 5: furosemide > 20 mg), the results were similar in relation to hemoglobin and BUN (fully adjusted model; *β* -0.03; 95% CI -0.04, -0.03; *P <*0.001) and BUN residual (*β* -0.03; 95% CI -0.04, -0.02; *P <*0.001).

In line 376, “Result: BUN and anemia development” paragraph page 24

When adjustment was done for the types and dose of diuretics and not just the use of diuretics (group 1: none; group 2: thiazide ≤ 12.5 mg, indapamide ≤ 1.5 mg, metolazone ≤ 2.5 mg; group 3: thiazide > 12.5 mg, indapamide > 1.5 mg, metolazone > 2.5 mg; group 4: furosemide ≤ 20 mg, torasemide ≤ 10 mg; group 5: furosemide > 20 mg), the effect of BUN on anemia development was similar (HR, 1.02: 95% CI 1.01, 1.04; *P*=0.002).

In line 428, “Result: BUN residual and anemia development” paragraph page 26

Similar results were obtained after adjustment for the types and dose of diuretics and not just the use of diuretics (HR, 1.02; 95% CI 1.00, 1.04; *P*=0.036).

***3. BUN residual is not easy to get during daily medical practice. If physicians cannot get the BUN residual easily, how to expand its clinical usage?***

Response: Thank you for your comment.

We fully agree with the reviewer’s opinion that BUN residual is difficult to get easily during daily medical practice. We did not try to emphasize the need to keep track of BUN residual, however, we wanted to present BUN residual to suggest that BUN is related to anemia as independent of the kidney function as possible. As we already mentioned that BUN residuals were derived from the distributions of eGFR and BUN of our study subjects, the value of BUN residuals would be different depending on the characteristics of subjects of different studies. However, the serial changes in BUN/eGFR ratio in stable non-dialysis CKD patients without intercurrent events that could alter tubular handling of urea and/or protein catabolism could be used to guess the changes in protein intake of specific patients.

***4. Table 5 dose not list the reference group.***

Response: Thank you for your comment.

We performed subgroup analysis according to the CKD stage and analyzed BUN, eGFR, and BUN residual as continuous variables. To clarify the meaning, we added a description to the manuscript.

In line 448, “Result: Discordant effects of BUN, BUN residual, and eGFR according to CKD stage” paragraph page 28

We performed subgroup analysis to show discordant effects of BUN, BUN residual, and eGFR according to the CKD stage (Table 5). BUN, eGFR, and BUN residential were analyzed as continuous variables.

**Reviewer #2:**

***1. In the Introduction, there is a sentence “no specific uremic toxin(s) responsible for anemia of 90 CKD has not been identified yet”. I guess “not” is redundant.***

Response: Thank you for your meticulous comment.

We revised our manuscript in the introduction

In line 90, “Introduction” paragraph page 5

In this regard, it appears uremic toxins might influence anemia development in CKD patients although no specific uremic toxin(s) responsible for anemia of CKD has ~~not~~ been identified yet.

***2. Although mentioned in the main text of Results, the proportions of patients receiving iron therapy or ESA should be added in Tables 1 and 2.***

Response: Thank you for your comment.

We already described the proportion of patients receiving iron therapy or ESA in Tables 1 and Table 3 in our previous manuscript along with hemoglobin value and anemia parameters. (Is not the reviewer's opinion about Table 3, not Table 2?)

In line 261, “Table 1” page 13

**Table 1.** Clinical characteristics of the study subjects at enrollment stratified by BUN levels.

| Characteristics | Total  (N = 2,196) | Blood urea nitrogen | | | | *P*-value |
| --- | --- | --- | --- | --- | --- | --- |
|  |  | 1st quartile | 2nd quartile | 3rd quartile | 4th quartile |  |
|  |  | (5.0-17.0 mg/dL) | (17.1-23.9 mg/dL) | (24.0-35.0 mg/dL) | (35.1-112.0 mg/dL) |  |
|  |  | (n = 572) | (n = 520) | (n = 557) | (n = 547) |  |
| Hemoglobin (g/dL) | 12.8 ± 2.0 | 14.0 ± 1.6 | 13.6 ± 1.8 | 12.5 ± 1.9 | 11.2 ± 1.5 | <0.001 |
| Iron (ug/dL) | 92.6 ± 35.1 | 102.1 ± 39.2 | 96.4 ± 34.4 | 88.7 ± 33.4 | 83.2 ± 29.9 | <0.001 |
| Ferritin (pmol/L) | 99.7 (53.7, 176.3) | 88.8 (42.9, 161.4) | 91.6 (53.3, 174.4) | 96.9 (58.9, 176.6) | 115.2 (63.7, 196.0) | <0.001 |
| TSAT (%) | 31.7 ± 12.1 | 32.9 ± 13.2 | 32.0 ± 11.7 | 31.2 ± 11.7 | 30.6 ± 11.4 | 0.011 |
| Anemia (%) | 970 (44.2) | 75 (13.1) | 137 (26.3) | 303 (54.4) | 455 (83.2) | <0.001 |
| Iron deficiency, n (%) | 1183 (54.9) | 316 (56.7) | 292 (27.4) | 308 (56.3) | 267 (49.4) | <0.001 |
| ESA use, n (%) | 167 (7.6) | 0 (0) | 4 (0.8) | 32 (5.8) | 131 (24.0) | <0.001 |
| Iron supplement, n (%) | 322 (14.7) | 20 (3.5) | 49 (9.5) | 85 (15.4) | 168 (30.8) | <0.001 |

In line 360, “Table 3” page 21

**Table 3.** Clinical characteristics of the study subjects with respect to anemia development**.**

| Characteristics | Total  (N = 1,169) | Groups | | *P*-value |
| --- | --- | --- | --- | --- |
|  |  | Incident anemia (-)  (n = 755) | Incident anemia (+)  (n = 414) |  |
| Hemoglobin (g/dL) | 14.2 ± 1.3 | 14.6 ± 1.3 | 13.5 ± 1.0 | <0.001 |
| Iron (ug/dL) | 102.3 ± 35.8 | 105.3 ± 35.7 | 96.8 ± 35.3 | <0.001 |
| Ferritin (pmol/L) | 99.3 (55.0, 174.4) | 105.7 (56.2, 175.4) | 89.8 (52.6, 170.7) | 0.112 |
| TSAT (%) | 33.5 ± 12.0 | 34.0 ± 11.9 | 32.6 ± 12.3 | 0.051 |
| Iron deficiency, n (%) | 617 (53.6) | 373 (50.3) | 244 (59.7) | 0.002 |
| ESA use, n (%) | 17 (1.5) | 1 (0.1) | 16 (3.9) | <0.001 |
| Iron supplement, n (%) | 50 (4.3) | 8 (1.1) | 42 (10.2) | <0.001 |

***3. Model 4 were adjusted for several additional variables including ESA. What is the criteria of ESA prescription? According to KDIGO guideline 2012 for anemia, 3.4.1: For adult CKD ND patients with Hb concentration >= 10.0 g/dl, we suggest that ESA therapy NOT be initiated. (2D) A lower Hb should be detected prior to ESA prescription, suggesting that the primary outcome, the development of anemia (Hb <13 for men and Hb <12 for women), occurred prior to ESA prescription. In this condition, why ESA is a concern and required to be adjusted?***

Response: Thank you for your valuable comment.

The indication of ESA prescription is as follows and added in method section.

In line 181, “Methods: Clinical and laboratory measurements” paragraph page 9

Erythropoiesis-stimulating agent (ESA) was prescribed under the discretion of physicians in participating hospitals in patients with anemia of CKD with hemoglobin < 10 g/dL in accordance with the KDIGO guidelines [30], excluding other causes of anemia. In addition, ESA was started in patients with eGFR < 30 mL/min/1.73m^2^ in accordance with the insurance coverage of Korea. During the use of ESA, physicians adjusted the dose of ESA to maintain hemoglobin at 10-11 g/dL (or hematocrit 30-33%) and stopped ESA if hemoglobin was > 11 g/dL (or hematocrit > 33%).

In accordance with your suggestion that ESA use itself is actually equal to the development of new anemia, we performed the analysis with adjustment excluding ESA use in which the association between anemia development and BUN (HR, 1.02, 95% CI 1.01, 1.04; *P*=0.001) or BUN residual (HR, 1.02, 95% CI 1.00, 1.04; *P*=0.027) showed similar results to the previous analysis. However, ESA variables were adjusted considering that ESA use could affect iron metabolism, inflammation, *etc*., and affect the main outcome, anemia, in this study, although we agreed with your suggestions.

***4. Another important issue is that the primary outcome, the occurrence of anemia, is a condition confounded by renal function at that time. As shown in Table 3, patients having incident anemia had higher Cre, lower eGFR, and higher CKD stages. To avoid this confounding, the authors should, for example, look at the proportion of anemia or Hb levels when entering CKD stage 4 or 5, or adjust for renal function when the primary outcome occurred (not simply adjusted for the baseline renal function).***

Response: Thank you for your comment.

We reanalyzed adjustment with renal function when anemia develops, not the baseline kidney function. Last follow-up eGFR was used for patients who did not develop anemia. The HR of anemia development for every 1 mg/dL increase in the BUN level was 1.04 (95% CI 1.02, 1.05; *P*<0.001). The result was similar to the previous analysis. However, in this case, changes in BUN and other variables are not reflected, and the purpose of this study is to understand the impact of baseline BUN on anemia and anemia development. In addition, we calculated BUN residual to minimize the impact of kidney function in BUN. Therefore, we want to maintain the current analysis.

***5. The sentence below the subtitle of “BUN residual and anemia development”, I think it’s “0.5 x SD of BUN” rather than “0.5 x SD of BUN residual”?***

Response: Thank you for your meticulous and helpful comment.

The previous manuscript had an error and we revised our manuscript.

In line 419, “Results: BUN residual and anemia development” paragraph page 26

The patients were categorized based on 0.5 × SD of BUN ~~residual~~ from fitted curve as shown in Figure 2.

***6. Is there statistical evidence to support the use of 0.5 SD as a criterion of fitting the curve? or it’s simply empirical?***

Response: Thank you for your comment.

Contrary to BUN which is actually measured value, BUN residual was derived from the distribution of BUN values on the same eGFR value. Therefore, we judged that the use of SD would be more appropriate than use of quartile grouping. The choice of 0.5 SD rather than 1 or 2 SD was determined simply to avoid excessive discrepancy in sample numbers in each group.

***7. The problems of discordant effects of BUN, BUN residuals, and eGFR as per the CKD stage shown in Table 5 were again confounded by renal function at the time of anemia developed.***

Response: Thank you for your comment.

We think that this comment is essentially similar to comment 4. Please refer to our response to comment 4. Again, we absolutely agree with the importance of the adjustment by the eGFR at the time of anemia development. In this regard, Cox regression using time-dependent variables for BUN, eGFR and other variables related to anemia would be more appropriate than the current analysis to evaluate the association between BUN and anemia. But increasing number of missed values during follow-up and different numbers of repeated measurement between variables (for example, hepcidin was measured only one time at the baseline and not repeatedly measured during follow-up) and other logistic problems would be expected. Anyway, it is still worth trying analysis with emphasis on adjustment by eGFR at the time of anemia development, which would be an objective of our next study.

***8. The authors used single measurement of BUN for analysis; however, several conditions may influence BUN levels, particularly daily protein intake and a relatively dehydration status after more than 8-hour fasting for the blood test. Did the authors consider to average the BUN levels within a given period of time for a better representation of the actual situation?***

Response: Thank you for your valuable comment. We thought that this

As the reviewer mentioned, BUN can be influenced by several factors, including volume status or medications. However, our cohort excluded those with acute illness at the time of study enrollment. In addition, patients have mostly prescribed diuretics for blood pressure control rather than edema, and the actual dose of the diuretic was not so high. Furthermore, the main exposure risk of this study is baseline BUN, which was not repeatedly measured (at short interval for example daily) in baseline, and sampling was conducted every six months to a year. Therefore, we want to maintain the current analysis.
