# Supplementary material for: Effects of blood urea nitrogen independent of the estimated glomerular filtration rate on the development of anemia in non-dialysis chronic kidney disease: The results of the KNOW-CKD study
Source: PLoS One. 2021 Sep 10;16(9):e0257305. doi: 10.1371/journal.pone.0257305 (PMC8432877; doi:10.1371/journal.pone.0257305)
Supplement: Attachment — Submitted filename: Response letter_R2_20210715_v3.1.docx [file pone.0257305.s006.docx]

**Point-by-Point Responses to the Reviewers**

**[Journal Requirements]**

Please review your reference list to ensure that it is complete and correct. If you have cited papers that have been retracted, please include the rationale for doing so in the manuscript text, or remove these references and replace them with relevant current references. Any changes to the reference list should be mentioned in the rebuttal letter that accompanies your revised manuscript. If you need to cite a retracted article, indicate the article’s retracted status in the References list and also include a citation and full reference for the retraction notice.

Response: The following one reference was additionally cited. However, the total number of references has not changed since the reference has been already cited for the criteria for the usage of ESA in the previous version of the manuscript.

In line 179, “Methods: Clinical and laboratory measurements” paragraph page 9

Intravenous or oral iron supplementation was prescribed under the discretion of physicians in participating hospitals if physicians judged it necessary to raise the hemoglobin of his patient or to reduce the dose of ESA in accordance with the KDIGO guidelines [30].

**[Additional Editor Comments]**
There are still several excellent comments and suggestions from Reviewer 2. ESA and iron treatment are important issues for analysis in this study. Besides, the statistical methods to fit the curve between eGFR and BUN should be described clearly. Please revise accordingly.

Response: Thank you for your opportunity. The manuscript was revised according to reviewer 2's comments.

**Reviewer #2:**

Kim et al. conducted a study evaluating the effects of BUN (and its residual) on anemia in non-dialysis CKD patients. Although interesting, a number of questions and concerns remained unanswered.

***1. In the Method section, the statistical methods (or equations) that used to fit the curve between eGFR and BUN should be disclosed, since BUN residual is an important factor discussed throughout the article.***

Response: Thank you for your meticulous comment. The manuscript was revised in method section.

In line 216, “Methods: Statistical analyses” paragraph page 11

To remove the effect of eGFR on BUN, a fitted curve between eGFR and BUN was obtained using the “splines” packages in R Statistics (version 4.04) which calculated the expected BUN value corresponding to eGFR using prediction function. The BUN residual was obtained by the vertical distance from each BUN value to the fitted curve between eGFR and BUN (Fig 2).

***2. To better illustrate the dose-dependent effects of BUN on variables in Table 1, did the authors consider the p-value for trend instead of just p-value?***
Response: Thank you for your comment.
The *P*-value for the trend and analysis method were added in Table 1.

***3. Based on the description in the article, ESA was prescribed in patients with renal anemia with hemoglobin < 10 g/dL in accordance with the KDIGO guidelines. The authors did not disclose the indication of iron supplementation. Besides iron deficiency based on iron profile, we usually prescribe iron only when Hb falls below certain level. In my opinion, those patients on ESA and iron treatment should still be removed from the second part of analysis because their Hb levels had already dropped earlier and against the notion to detect de novo anemia. The patients remained non-anemic by definition during follow-up could simply be owing to escalated dose of ESA and iron, which could possibly offset the effect of increased BUN. If the authors decided not to change the current analysis, at least the description in the responding to reviewers’ comments “we performed the analysis with adjustment excluding ESA use in which the association between anemia development and BUN (HR, 1.02, 95% CI 1.01, 1.04; P=0.001) or BUN residual (HR, 1.02, 95% CI 1.00, 1.04; P=0.027) showed similar results to the previous analysis” should be added in the article.***

Response: Thank you for your valuable comment.

The indication of iron supplementation was added in the method section.

In line 179, “Methods: Statistical analyses” paragraph page 9

Intravenous or oral iron supplementation was prescribed under the discretion of physicians in participating hospitals if physicians judged it necessary to raise the hemoglobin of his patient or to reduce the dose of ESA in accordance with the KDIGO guidelines [30].

But in real world clinical practice, there were inevitable variations or deviations from accepted guideline. In only one patient in which ESA was prescribed, ESA was prescribed only once at enrollment when hemoglobin value was 14.8 g/dL. This patient remained non-anemic without ESA during the entire follow up period of more than 3 years. All eight patients with iron prescription, ESA was not concurrently used and their eGFR values were 28.7, 51.7, 64.9, 83.0, 87.8, 71.3,61.8, and 65.6 ml/min/1.73m^2^ respectively, which suggest the low probability of the presence of anemia of CKD in these patients.

However, we could not totally exclude the possibility of misassignment of these patients to group without incident anemia as you pointed out and we performed further analysis as below.

The associations between anemia development and BUN or BUN residual were further analyzed by multivariable analysis, excluding iron replacement therapy and ESA in model 4 (model 5). In addition, sensitivity analysis was performed except for patients in the non-incident anemia group who were prescribed iron replacement therapy or ESA at enrollment. The association between anemia development and BUN or BUN residual presented similar results to the previous analysis.

In line 381, “Results: BUN and anemia development” paragraph page 26

To remove the effect of possible misassignment of subjects using ESA or iron to group without incident anemia, analysis excluding iron replacement therapy and ESA use in model 4 (model 5) was performed, which showed the association between anemia development and BUN (HR, 1.03, 95% CI 1.01, 1.04; *P* <0.001) similar to model 4 (Table 4). In another way, sensitivity analysis was performed excluding 9 patients in the non-incident anemia group who were prescribed iron replacement therapy or ESA at enrollment without adjustment by iron replacement therapy and ESA use. The association between anemia development and BUN (HR, 1.03, 95% CI 1.01, 1.04; *P* <0.001: model 5) presented similar results.

In line 440, “Results: BUN residual and anemia development” paragraph page 28

To remove the effect of possible misassignment of subjects using ESA or iron to group without incident anemia, analysis excluding iron replacement therapy and ESA use in model 4 (model 5) was performed, which showed the association between anemia development and BUN residual (HR, 1.02, 95% CI 1.00, 1.04; *P*=0.035) similar to model 4 (Table 4). In another way, sensitivity analysis was performed excluding 9 patients in the non-incident anemia group who were prescribed iron replacement therapy or ESA at enrollment without adjustment by iron replacement therapy and ESA use. The association between anemia development and BUN residual (HR, 1.02, 95% CI 1.00, 1.04; *P=* 0.027: model 5) presented similar results.

***4. As disclosed in the Methods, the study cohort enrolled about 600 subjects with polycystic kidney disease. How many patients had polycystic kidney disease in this study? Anemia is on average less severe in ADPKD patients.***
Response: Thank you for your helpful comment.

There were 362 (16.5%) ADPKD patients and hemoglobin levels according to cause of CKD were as follows. Mean hemoglobin levels in ADPKD patients were 13.2 ± 1.8 mg/dL. The hemoglobin levels in ADPKD were higher than in diabetic nephropathy patients, similar to other causes of CKD.

| Cause of CKD | Hemoglobin level (g/dL) |
| --- | --- |
| Glomerulonephritis | 13.2 ± 1.9 |
| Diabetic nephropathy | 11.7 ± 1.8 |
| Hypertensive nephropathy | 13.3 ± 2.0 |
| ADPKD | 13.2 ± 1.8 |
| Others | 12.9 ± 1.2 |

Causes of CKD were added in Table 1 and 3.

***5. In a paragraph evaluating the dose of diuretics, which kind of thiazide did the authors refer to? Thiazides actually represent a group of agents, such as hydrochlorothiazide and chlorothiazide. And according to WHO Collaborating Centre for Drug Statistics Methodology (***[***https://www.whocc.no/atc_ddd_index/?code=C03BA&showdescription=no***](https://www.whocc.no/atc_ddd_index/?code=C03BA&showdescription=no)***), the DDDs for indapamide and metolazone are 2.5 mg and 5 mg. What’s the rationale of 1.5 mg for indapamide and 2.5 mg for metolazone in groups 2 and 3?***

Response: Thank you for your valuable comment.

Thiazide was all hydrochlorothiazide, and the manuscript and table were modified to clarify the meaning.

The commercialized dose of indapamide in Korea is 1.5mg or 2.5mg per tablet. In the present study, all patients prescribed indapamide took single 1.5 mg tablet. Therefore, 1.5 mg for indapamide and 2.5 mg for metolazone were categorized in the same group although there is some discrepancy in DDD as you pointed out.

***6. Is it possible that low BUN residual and low DPI in patients with de novo anemia shown in Table 3 simply suggested that these patients had malnutrition?***

Response: Thank you for your valuable comment.

As this study is an observational study, it is difficult to establish an exact causal relationship. There are no definitive data to explain the causes of low BUN retention and low DPI. Malnutrition and/or inflammation could be one of plausible explanation.

***7. In Table 5, I am not sure how the statistical process was performed. Did the authors repeat the regression analysis in each CKD stage for the effects of BUN and BUN residual on anemia development? If so, there’s only 109 patients in CKD stage 4-5, and over-adjustment for variables is a concern.***

Response: Thank you for your meticulous comment.

As the reviewer said, the regression analysis was performed in each CKD stage for BUN and BUN residual effects on anemia development. Although there is some over-adjustment, the same model was inevitably used for subgroup comparison.

Even when the analysis in the CKD stage 4-5 group was performed by the adjustment with reduced number of variables as below, the results were similar to those of the previous analysis.

| **CKD stage** | **Model A** | | | | **Model B** | | | |
| --- | --- | --- | --- | --- | --- | --- | --- | --- |
|  | **BUN** | | **eGFR** | | **BUN residual** | | **eGFR** | |
|  | **HR (95% CI)** | ***P*** | **HR (95% CI)** | ***P*** | **HR (95% CI)** | ***P*** | **HR (95% CI)** | ***P*** |
| **Stage 4-5**  (n = 109) | 1.01  (0.98, 1.04) | 0.610 | 0.98  (0.93, 1.04) | 0.561 | 1.00  (0.97, 1.04) | 0.784 | 0.97  (0.93, 1.02) | 0.279 |

Model A: Adjusted for BUN, age, sex, eGFR, UPCR, DM, CCI, log CRP albumin TSAT ferritin.

Model B: Adjusted for BUN residual instead of BUN in model A.

BUN, blood urea nitrogen; HR, hazard ratio; CI, confidence interval; DM, diabetes mellitus; CCI, Charlson comorbidity index; eGFR, estimated glomerular filtration rate as determined by the CKD-EPI creatinine equation; UPCR, urinary protein-to-creatinine ratio; CRP, C-reactive protein; TSAT, transferrin saturation

In line 559, “Discussion” paragraph page 36

Because the number of CKD stage 4-5 group patients was smaller than that of other CKD stages, it should be noted that too many variables compared to the number of patients could overly adjust the analysis in these stage groups. However, when multivariable correction factors were reduced for analysis (adjusted for BUN (or BUN residual), age, sex, eGFR, UPCR, DM, CCI, log CRP, albumin, TSAT, and ferritin), there was no significant association of BUN, BUN residual, and eGFR with anemia development.

***8. In the Discussion, a sentence goes “The National Cooperative Dialysis Study demonstrated that patients with higher midweek pre-dialysis BUN resulting from inadequate dialysis dose to handle their protein intake had a higher failure rate [38].” What kind of failure did the authors refer to?***

Response: Thank you for your valuable comment.

The manuscript was revised in the discussion section.

In line 571, “Discussion” paragraph page 36

The National Cooperative Dialysis Study demonstrated that patients ~~with higher midweek pre-dialysis BUN resulting from~~ receiving inadequate dialysis dose to handle their protein intake had a higher failure rate of the attaining the target midweek pre-dialysis BUN [38].
